# Patient iPSC-derived neural progenitor cells display aberrant cell cycle control, p53, and DNA damage response protein expression in schizophrenia

**DOI:** 10.1186/s12888-024-06127-x

**Published:** 2024-10-31

**Authors:** Aaron Stahl, Johanna Heider, Richard Wüst, Andreas J. Fallgatter, Katja Schenke-Layland, Hansjürgen Volkmer, Markus F. Templin

**Affiliations:** 1 Institute of Biomedical Engineering, Department for Medical Technologies and Regenerative Medicine, University of Tübingen, Tübingen, 72076 Germany; 2 NMI Natural and Medical Sciences Institute at the University of Tübingen, Markwiesenstraße 55, Reutlingen, 72770 Germany; 3 Department of Psychiatry, Tübingen Center for Mental Health (TüCMH), University of Tübingen, Tübingen, 72076 Germany; 4 German Center for Mental Health (DZPG), Partner Site Tübingen, Tübingen, 72076 Germany

**Keywords:** Proteomics, DigiWest, IPSC, Neural progenitors, Schizophrenia, Cellular signaling, Cell cycle, p53

## Abstract

**Background:**

Schizophrenia (SCZ) is a severe psychiatric disorder associated with alterations in early brain development. Details of underlying pathomechanisms remain unclear, despite genome and transcriptome studies providing evidence for aberrant cellular phenotypes and pathway deregulation in developing neuronal cells. However, mechanistic insight at the protein level is limited.

**Methods:**

Here, we investigate SCZ-specific protein expression signatures of neuronal progenitor cells (NPC) derived from patient iPSC in comparison to healthy controls using high-throughput Western Blotting (DigiWest) in a targeted proteomics approach.

**Results:**

SCZ neural progenitors displayed altered expression and phosphorylation patterns related to Wnt and MAPK signaling, protein synthesis, cell cycle regulation and DNA damage response. Consistent with impaired cell cycle control, SCZ NPCs also showed accumulation in the G2/M cell phase and reduced differentiation capacity. Furthermore, we correlated these findings with elevated p53 expression and phosphorylation levels in SCZ patient-derived cells, indicating a potential implication of p53 in hampering cell cycle progression and efficient neurodevelopment in SCZ.

**Conclusions:**

Through targeted proteomics we demonstrate that SCZ NPC display coherent mechanistic alterations in regulation of DNA damage response, cell cycle control and p53 expression. These findings highlight the suitability of iPSC-based approaches for modeling psychiatric disorders and contribute to a better understanding of the disease mechanisms underlying SCZ, particularly during early development.

**Supplementary Information:**

The online version contains supplementary material available at 10.1186/s12888-024-06127-x.

## Background

Schizophrenia spectrum disorders (SCZ) are a group of severe neurodevelopmental disorders with a highly heterogeneous clinical presentation of a broad range of symptoms including hallucinations, delusions, emotional blunting and cognitive deficits [1]. Current antipsychotic treatment options for SCZ are purely symptomatic and show only limited efficacy in alleviating negative and cognitive symptoms [2, 3]. Hence, there is an urgent need to develop causal treatments, which has so far been hampered by the still limited knowledge of the molecular mechanisms that are involved in the disease. As SCZ is regarded as a neurodevelopmental disorder, alterations occurring during early brain development are of particular interest. Genome-wide association and exome sequencing studies have linked > 100 risk loci to SCZ but information on the protein level remains limited [4, 5].

Induced pluripotent stem cells (iPSC) have emerged as a valuable source for phenotypic studies of disease-relevant cell types with a patient-specific genetic background. Transcriptomic analysis and shotgun proteomics of iPSC-derived neural progenitor cells (NPC) and neurons have previously helped to gain insight into potential disease-related processes. Most studies investigating the proteome in SCZ employ mass spectrometry approaches [6–8]. Deregulated expression of genes and proteins involved in protein synthesis, cell adhesion, regulation of the cytoskeleton, oxidative stress and neuronal differentiation have been identified in SCZ [8–11]. Additionally, deregulations in key signaling pathways have been described, among which the most prominent changes have been reported for proteins of the Wnt and Akt/ glycogen synthase kinase-3 beta (GSK3 beta) signaling pathways in both iPSC and patient studies [10, 12–16]. Postmortem studies have also linked the mitogen-activated protein kinase (MAPK)/ extracellular signal-regulated kinase (ERK) pathway to SCZ [17–19]. Despite the important insights that have been gained by these studies, many important questions regarding the connection between aberrant signaling and cellular (patho)-phenotypes remain unanswered. Moreover, with most studies focusing on the transcriptome level, proteomic studies remain underrepresented in the field. However, they are of great importance given that gene expression changes do not always translate to the protein level. Especially the study of phosphorylated proteins remains difficult using high throughput proteomic approaches but is of great importance for entangling mechanisms surrounding pathway activity. Applying such techniques to relevant neuronal cell types would greatly expand the knowledge of the biological processes at play during the early phases of disease development.

Here, we employ high-throughput Western Blotting (DigiWest) to study protein expression and phosphorylation of 133 proteins in SCZ patient-derived cells on the iPSC and NPC level in a targeted proteomics approach. The DigiWest is a multiplexed Western Blot derivative which transfers the Western Blot onto a bead-based system, thereby greatly increasing throughput while retaining the sensitivity of traditional Western Blotting. In this fashion, expression data from up to 200 proteins and phosphoproteins can be obtained in a targeted manner, allowing extensive analysis of cellular signaling pathways [20]. Applying this method, we aimed to compare differences in protein expression patterns between control (CTR) and SCZ in iPSC and NPC, respectively, to identify potential disease-relevant alterations in cellular signaling.

We report several aberrant SCZ-specific protein signatures, exclusive to NPC, with regards to signaling pathway activity. Most notably, we found proteomic alterations in cell cycle control, DNA-damage response regulation along with impaired differentiation capacity of SCZ NPC. Furthermore, we were able to correlate these alterations to p53 expression and phosphorylation levels in SCZ-derived cells, which provides further mechanistic insights into the early developmental stages of SCZ.

## Methods

### iPSC line information and maintenance

For detailed information on the three control and four patient-derived iPSC lines used see Additional file 1—Table S1. The CTR1 iPSC line was purchased from Thermo Fisher Scientific (Waltham, MA, USA—#A18945). The CTR2 iPSC line was a gift from the Tumorbiology group at NMI Reutlingen. For clinical patient data, see [21]. iPSC were maintained on plates coated with hESC-qualified Matrigel (Corning, Corning, NY, USA) in mTeSR Plus (STEMCELL Technologies, Vancouver, Canada). For single cell seeding, iPSC were enzymatically passaged using Accutase (Sigma-Aldrich, St. Louis, MO, USA).

### NPC generation and maintenance

NPC were generated from iPSC following the embyroid body (EB) protocol of the STEMdiff™ SMADi Neural Induction Kit (STEMCELL Technologies) according to the manufacturer's instructions. The obtained NPC were cultured in STEMdiff™ Neural Progenitor medium (STEMCELL Technologies) on 6-well plates coated with 20% poly-l-ornithine (Sigma-Aldrich) for 2h at room temperature (RT) and 10 µg/ml Laminin (Sigma-Aldrich) at 37 °C overnight. NPC were enzymatically passaged with Accutase. For all experiments, only passage 2 NPC from three independent rounds of differentiation were used to ensure comparability of results.

### DigiWest protein profiling

iPSC were obtained in triplicate and NPC samples from three independent differentiations. DigiWest was performed as published [20] using 12 µg of cellular protein. In brief, the NuPAGE system (Life Technologies) was used for gel electrophoresis and blotting onto PVDF membranes. Proteins were biotinylated on the membrane using NHS-PEG12-Biotin (50 µM) in PBST for 1 h. Sample lanes were cut into 96 strips (0.5 mm each) and placed in one well of a 96-well plate before adding 10 µl elution buffer (8 M urea, 1% Triton-X100 in 100 mM Tris–HCl pH 9.5). Each strip/protein fraction was incubated with 1 distinct Neutravidin-coated MagPlex bead population (Luminex, Austin, TX, USA). Coupling was performed overnight, and non-bound binding sites were blocked with 500 µM deactivated NHS-PEG12-Biotin for 1 h. By pooling all 96 protein-loaded bead populations, the original sample lane was reconstituted.

5 µl aliquots of bead mix were added to 96-well plates containing 50 µl assay buffer (Blocking Reagent for ELISA (Roche) supplemented with 0.2% milk powder, 0.05% Tween-20 and 0.02% sodium azide). Upon discarding of the assay buffer, 30 µl of primary antibody (diluted in assay buffer) was added per well. After overnight incubation at 15 °C, the bead-mixes were washed twice with PBST and species-specific PE-labelled (Phycoerythrin) secondary antibodies (Dianova, Hamburg, Germany) were added for 1 h at 23 °C. Beads were washed twice with PBST before readout on a Luminex FlexMAP 3D instrument.

One hundred thirty-seven primary antibodies (Additional file 2) were selected from a collection of > 1 500 available antibodies, all of which are performance-evaluated and routinely used in DigiWest. Selection was largely based on covering signaling pathways/cellular functions for which associations with SCZ have been described in previous genetic/transcriptomic/proteomic studies as mentioned elsewhere in this manuscript Refs [8–19, 22, 23]. Likewise, to accurately monitor pathway activity during neuronal differentiation, signaling proteins from neurodevelopmental pathways (Wnt [24], Hippo [25], Hedgehog [26], Smad [27] signaling) were additionally selected along with other common signaling and marker proteins. Pathway allocation of analytes was mapped based on the Kyoto Encyclopedia of Genes and Genomes (KEGG) database [28, 29].

For peak integration, an Excel-based analysis tool was used. A total of 148 peaks were identified, with 133 (89.9%) generating reliable signals. Signal intensity was separately normalized to total protein amount on the beads within each differentiation. The software package MeV 4.9.0 was used for heatmap generation and statistical analysis [30]. For heatmaps, fluorescent signals were either median centered across samples for a given analyte (iPSC versus NPC) or centered around the average signal of the three CTR lines within each differentiation (CTR versus SCZ) before Log2 transformation. Raw and normalized DigiWest data can be found in Additional file 3 and 4, respectively.

### Immunocytochemistry

For immunocytochemical staining, iPSC or passage-matched NPC were seeded on 96-well µClear™ plates (Greiner, Kremsmünster, Austria). Once 70–80% confluence was reached, cells were fixed with 4% PFA in PBS for 15 min at RT. Cells were washed 3 × with PBS and incubated with PBS + 1 × BMB blocking reagent (Roche) + 0.1% Triton (Carl Roth, Karlsruhe, Germany) for 30 min at RT. Primary antibodies were diluted in blocking/permeabilization solution and incubated overnight at 4 °C. Afterwards, cells were washed 3 × with PBS. Secondary antibodies were incubated in blocking/permeabilization solution for 2h at RT on an orbital shaker and washed with PBS. For nuclear staining, Hoechst 33,258 (Sigma-Aldrich) was diluted in PBS and incubated for 30 min at RT. A complete list of ICC antibodies used in this study can be found in Additional file 1 – Table S2.

### Image acquisition and analysis of immunocytochemical staining

**Fig. 1:**
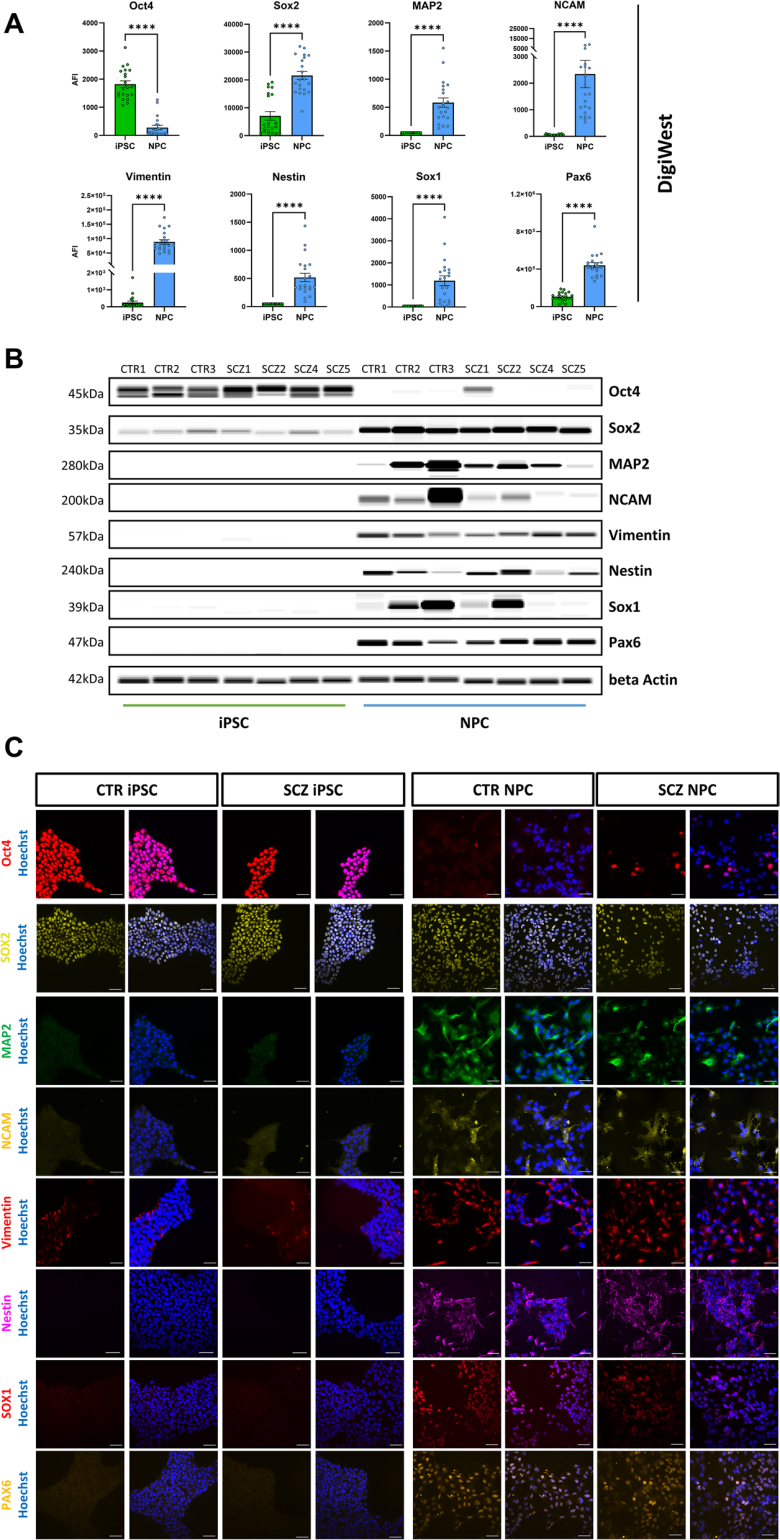
Marker expression in iPSC and NPC. **A**: DigiWest data (AFI = accumulated fluorescent intensity) of cell-type specific marker proteins in iPSC (*n* = 21) and NPC (*n* = 21); Mann–Whitney test. **B**: Western Blot mimic (gray-scale image) of markers displayed in A (exemplarily shown for one differentiation only). **C:** Example ICC images of marker expression in iPSC (left) and NPC (right) obtained by high-content microscopy. Scale bars: 50 µm. CTR and SCZ cells will be addressed separately at a later stage. *p < 0.05, ****p < 0.0001. Error bars: S.E.M

**Fig. 2:**
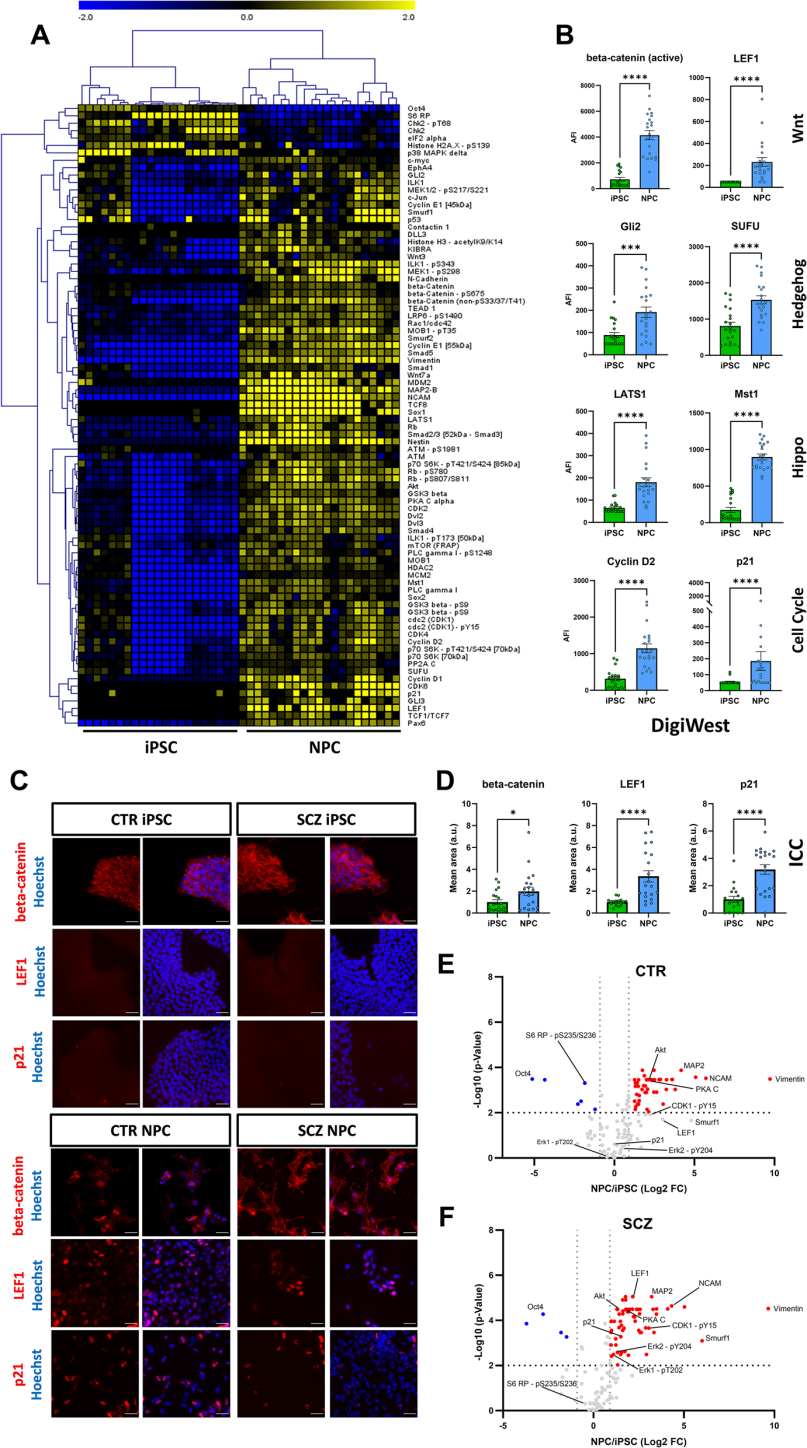
Expression signatures and pathway upregulation during differentiation. **A**: Heatmap and Hierarchical Cluster analysis of analytes significantly different between iPSC (*n* = 21) and NPC (*n* = 21) samples (Wilcoxon test, *p* < 0.001). **B**: DigiWest data (AFI = accumulated fluorescent intensity) for a subset of proteins with differential expression in iPSC (*n* = 21) and NPC (*n* = 21). Proteins are grouped according to their pathway allocation; Mann–Whitney test. **C**: Example ICC images of beta-catenin, LEF1 and p21 expression in iPSC (top) and NPC (bottom). Scale bars: 50 µm. **D**: Quantified ICC signals of proteins exemplarily shown in C (iPSC *n* = 21, NPC *n* = 21) obtained by high-content microscopy. Data are shown relative to mean iPSC signal; Mann–Whitney test. **E–F**: Volcano plot of separate iPSC vs NPC comparison for CTR (**E**—iPSC *n* = 9, NPC *n* = 9) and SCZ (**F**—iPSC *n* = 12, NPC *n* = 12) samples (Wilcoxon-Test, *p* < 0.01). Significantly upregulated proteins are shown in red, downregulated proteins in blue (analytes with FCs < I1I are excluded). Analytes with a significant interaction effect between cell type and disease allocation (*p* < 0.05, 2-Way-ANOVA) are highlighted (also see Additional File 5 – Figure S6 and Additional File 1—Table S3). *p < 0.05, ***p < 0.001, ****p < 0.0001. Error bars: S.E.M

**Fig. 3:**
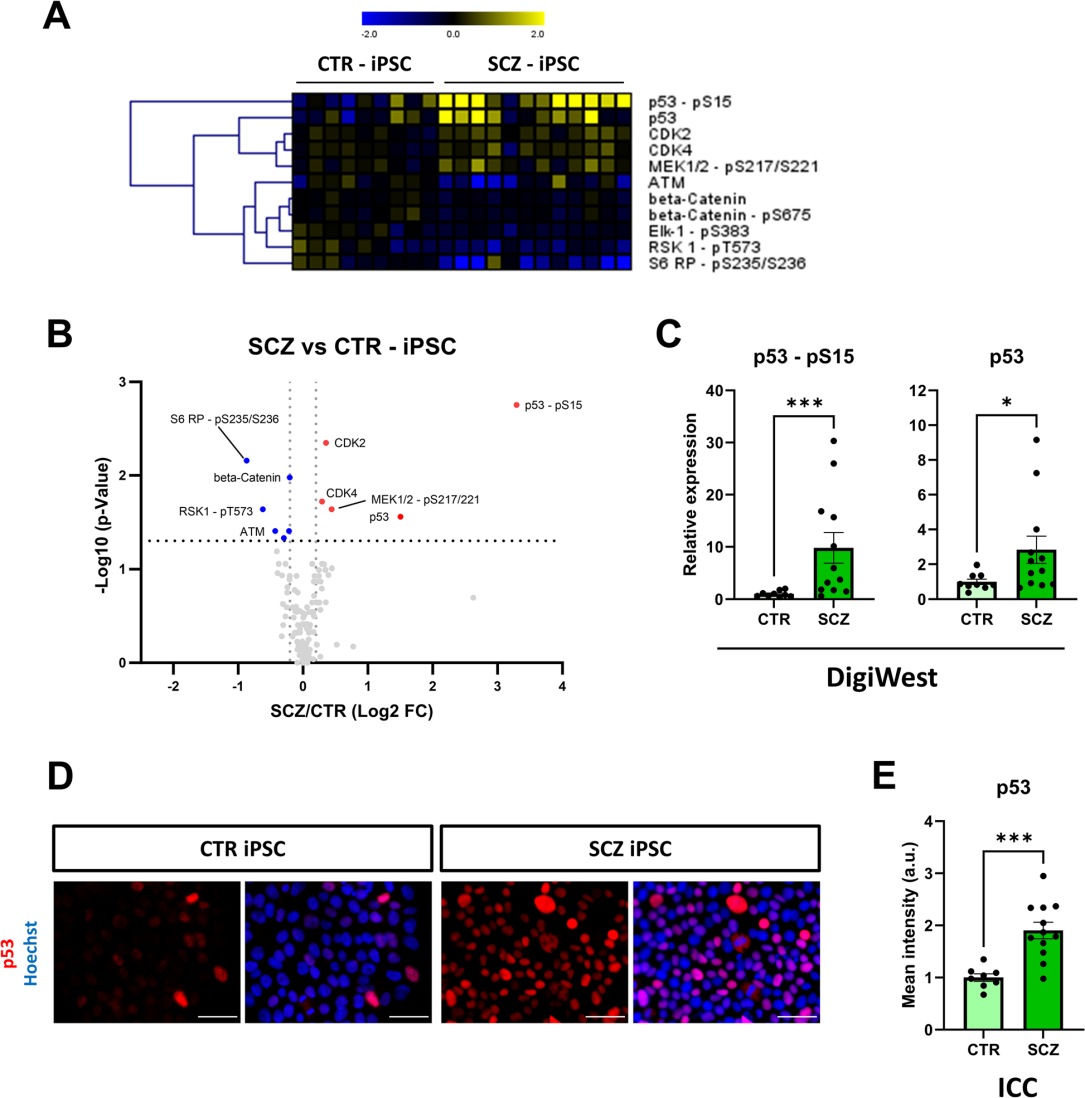
SCZ-specific alterations in iPSC. **A**: Heatmap with Hierarchical Cluster analysis (HCL) of analytes significantly different (Wilcoxon Test, *p* < 0.05) between CTR (*n* = 9) and SCZ (*n* = 12) iPSC. Log2-transformed data is shown relative to mean signal across CTR lines of the respective differentiation. **B**: Volcano plot of comparison shown in A. **C**: DigiWest data (relative to control mean) of p53 and p53 – pS15 expression comparing CTR (*n* = 9) and SCZ (*n* = 12) iPSC; Mann–Whitney test. **D**: Example images of total p53 ICC staining in iPSC obtained by high-content microscopy. Scale bars: 50 µm. **E**: Quantified ICC signal of total p53 expression (relative to CTR mean) in CTR (*n* = 9) and SCZ (*n* = 12) iPSC (unpaired t-test). *p < 0.05, ***p < 0.001. Error bars: S.E.M

**Fig. 4:**
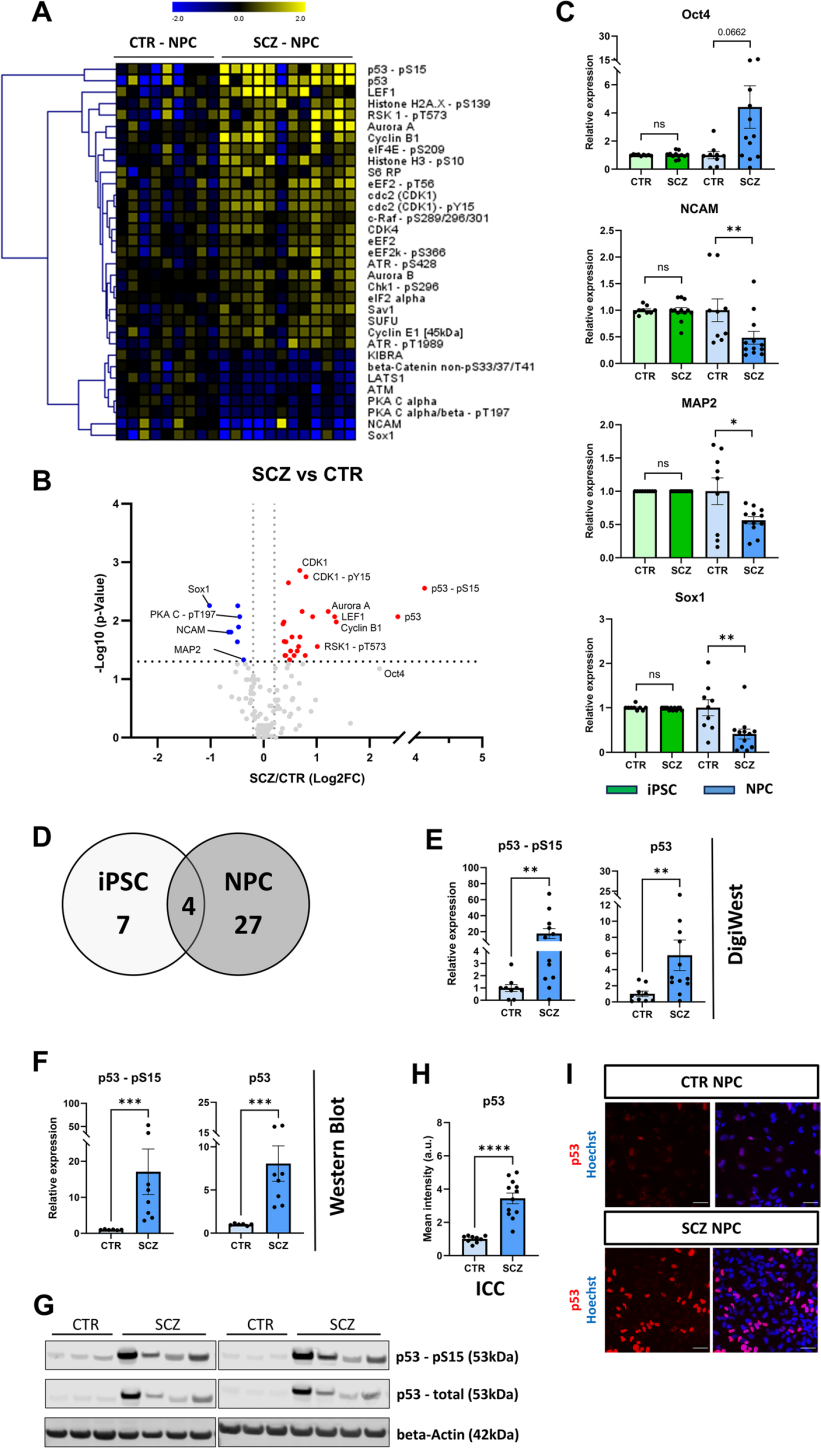
SCZ-specific alterations in NPC. **A**: Heatmap with Hierarchical Cluster analysis (HCL) of analytes significantly different (Wilcoxon Test, *p* < 0.05) between CTR (*n* = 9) and SCZ (*n* = 12) NPC. Log2-transformed data is shown relative to mean signal across CTR lines of the respective differentiation. **B**: Volcano plot of comparison shown in A. **C**: DigiWest data (relative to CTR mean) of Oct4, MAP2, NCAM and Sox1 expression in CTR (*n* = 9) and SCZ (*n* = 12) iPSC and NPC, respectively; Mann–Whitney test. **D**: Venn diagram showing the number of analytes differentially regulated between SCZ and CTR in the respective cell type. **E**: DigiWest data (relative to CTR mean) of p53 – pS15 and p53 (total) expression; CTR *n* = 9, SCZ *n* = 12, Mann–Whitney test. **F**: Quantified Western Blot signals of p53 – pS15 and p53 (total) expression in NPC (relative to CTR mean). Intensities were normalized to beta-Actin signal; CTR *n* = 6, SCZ *n* = 8, Mann–Whitney test. **G**: Western Blot images corresponding to quantification shown in F. **H**: Quantified ICC signal of total p53 expression (relative to CTR mean) in CTR (*n* = 9) and SCZ (*n* = 12) NPC (unpaired t-test). **I:** example images of total p53 ICC staining as obtained by high-content microscopy. Scale bars: 50 µm. *p < 0.05, **p < 0.01, ***p < 0.001, ****p < 0.0001. Error bars: S.E.M

Z-Stack images were acquired from four sites per well with the ImageXpress Micro Confocal High-Content Imaging System (Molecular Devices, San Jose, CA, USA). To ensure unbiased image acquisition and analysis, all acquisition parameters for individual antibodies were kept constant within replicates. For image analysis, the software MetaXpress (Molecular Devices) was used. 2D projections were generated and intensity/size thresholding (for nuclear and spot-like staining patterns) was applied to generate a mask covering the fluorescent signal for each marker. Depending on the marker expression pattern (nuclear vs. cytosolic), different parameters were used for analysis. For nuclear stains and stainings in which cells/nuclei were either positive or negative for a marker, the mean stained area was analyzed per image. For stainings, which were present in all cells but varied in intensity between different cells, the mean intensity per image was analyzed. In any case, values from one well were averaged and normalized to Hoechst signal to account for differences in cell density. Data were centered around the mean signal across either all iPSC (Figs. 1C, 2C) or all CTR clones (Figs. 3E, 4H, 6B). Data was obtained from three biological replicates.

### Flow cytometry cell cycle analysis

Passage-matched NPC were seeded on 12-well plates at an appropriate density to ensure exponential growth until fixation (8.5 × 10^4/cm^2^). The following day, NPC were treated with DMSO or Nocodazole (Sigma-Aldrich, 125nM) for 24h. Afterwards, the cells were detached, counted and 6 × 10^5 cells per condition fixed with 4% PFA for 15 min at RT. Cells were washed 3 × with PBS and treated with 100 µg/ml RNAse A (New England Biolabs, Ipswich, MA, USA) and 0.1% Triton X-100 in TrypLE express (Thermo Fisher) at 37 °C for 30 min for RNA removal and cell permeabilization. For fluorescence-activated cell sorting (FACS), 3 × 10^5 cells were transferred per well of a 96-well plate and stained with 2 µg/ml propidium iodide (PI) solution (BioLegend, San Diego, CA, USA) in TrypLE express for 30 min at 37 °C. Analysis of PI staining was performed using the FACS Fortessa™ Cell Analyzer (BD Biosciences, Franklin Lakes, NJ, USA). A minimum of 15,000 events was recorded per well, from two wells per condition. Analysis of FACS data was performed using FlowJo 10 (BD Biosciences). The gating strategy is outlined in Additional file 5 – Figure S11A. Cell cycle phases were identified using the build-in univariate cell cycle analysis tool (Dean-Jett-Fox model, [31]).

### Cell proliferation assay

Passage-matched NPC were seeded at a density of 2 × 10^4 cells/well in a 96-well plate in STEMdiff™ Neural Progenitor medium. For 72h, cells were cultivated in the IncuCyte® Live Cell Analysis System (Sartorius, Göttingen, Germany). Whole-well brightfield images were acquired in 4 h intervals with a 10 × objective in triplicates for each NPC line. Proliferation was analyzed using the IncuCyte® Basic Analyzer. Confluence was normalized to t = 0. Data was obtained from three biological replicates.

### Western blot

Size-separation via SDS-PAGE was performed as described above (DigiWest) using 10 μg of protein and blotted onto a Nitrocellulose membrane (VWR). Blocking was performed for 1 h using 5% milk powder (Roth) in TBST. Primary antibodies (identical to those used in DigiWest) were diluted in 1% BSA (Roth) in TBST and incubated overnight at 4 °C. Blots were washed 5 × for 5 min in TBST before adding fluorescently labelled secondary antibodies for 1 h (donkey anti-Rabbit IgG (H + L) coupled to IRDye 800CW (LI-COR, Lincoln, NE, USA) and donkey anti-Mouse IgG (H + L) coupled to IRDye 680CW (LI-COR), each at a dilution of 1:10,000 in 5% milk powder in TBST) and detected on a LI-COR instrument. Before analysis, blots were washed 5 × for 5 min in TBST and bands were quantified using Image Studio. Uncropped Western Blot images can be found in Additional file 5 – Figure S15.

### Neurite outgrowth assay

Passage-matched NPC were seeded into 48-well plates at 1 × 10^4^ cells per well in Neural Progenitor medium. After 24 h, medium was changed to N2-medium (t = 0 h), consisting of DMEM/F12 + 15 mM HEPES, 1 × N2-supplement, 1% non-essential amino acids, 1% GlutaMAX, 1.5% glucose solution (all Thermo Fisher), 10 µM SB431542 (Bio-Techne, Minneapolis, MN, USA), 1 µM XAV939 (Sigma-Aldrich), 100 nM LDN193189 (STEMCELL Technologies), 10 ng/ml BDNF (Thermo Fisher). From then on, cells were cultured in the IncuCyte® Live Cell Analysis System (Sartorius) and imaged every 4 h in brightfield mode at 10 × magnification. Two wells were imaged per line, and 4 sites per well. After 24 h, a complete medium change with N2 medium was performed and cells were cultured until t = 40 h without further medium changes. Using the IncuCyte® NeuroTrack analysis module (Sartorius), neurite length (in mm) per number of cell body clusters was analyzed for each time point. Analysis parameters were defined as follows: cell body clusters with a minimum area of 200 µm, containing cells with a minimum width of 7 µm, were counted. Neurite width was defined as 1 µm. These parameters were analyzed for each of the four sites imaged and then averaged per well. Data was obtained from four biological replicates. Values at t = 0 h were set to 1.

### Statistics

Statistical analysis was performed using GraphPad Prism 10 (Graphpad Software). Data was tested for normality using the Shapiro–Wilk Test. Only if both groups were normally distributed, groups were compared via unpaired, two-tailed t-test. If normality was not met, the two-tailed Mann–Whitney-U Test was used. For DigiWest data-based comparisons (used in heatmaps), the Wilcoxon-signed rank test was used. For neurite outgrowth and flow cytometry analysis, data was analyzed with a 2-way ANOVA and Tukey’s multiple comparisons test. N numbers and statistical details for each experiment can be found in the respective figure legend. In all cases, a *p*-value < 0.05 was considered significant unless stated otherwise.

## Results

### Characterization of developmental marker expression and signaling pathway activation during NPC differentiation via DigiWest

To characterize the differentiation process from iPSC into NPC, we examined the expression of 133 proteins from three healthy control lines and four SCZ patient lines. This set of proteins was composed of routinely used cell type markers for iPSC and NPC, as well as proteins involved in signaling pathways associated with neurodevelopment and previously linked to SCZ. First, we aimed to investigate the differences between the iPSC and NPC stage independent of disease allocation. Expression signatures of all measured proteins in NPC were consistent between three individual differentiations (Additional file 5 – Figure S1) where NPC of the respective differentiation do not cluster together, demonstrating high reproducibility. When comparing all iPSC (*n* = 21) and NPC (*n* = 21) samples, expectedly observed strong changes in the expression of cell-type specific markers. In NPC, DigiWest data shows downregulation of the pluripotency marker Oct4. Sox2 was expressed in both cell types but to a greater extent in NPC. Clear upregulation of neurodevelopmental/NPC-associated markers microtubule-associated protein 2 (MAP2), Vimentin, neural cell adhesion molecule (NCAM), paired-box protein Pax-6 (Pax6), SRY-Box Transcription Factor 1 (Sox1) and Nestin was observed (Fig. 1A, Additional file 5—Figure S2). Although variability in expression levels between individual patient-derived lines does persist, the two cell types are clearly distinguishable when displayed in a Western Blot-like format, as shown in Fig. 1B (Additional file 5 – Figure S3). Expression of these cell type-specific markers was confirmed by immunocytochemical staining (ICC) in both iPSC and NPC (Fig. 1C) with good comparability of DigiWest and ICC as exemplarily shown for four markers (Additional file 5 – Figure S4).

The high-throughput nature of DigiWest allowed us to assess expression of over 130 proteins and phosphoproteins and thus look beyond the expression of traditional cell type markers. We observed further pronounced and consistent expression differences between iPSC and NPC for various other proteins, leaving 61% (81/133) of analytes differentially expressed at a significance level of *p* < 0.001 (Fig. 2A, Additional file 5 – Figure S2), with the majority becoming strongly upregulated in NPC. These upregulations included key members of signaling pathways involved in neurodevelopment (Fig. 2B), such as Wnt signaling (active and total beta-catenin, Wnt3/7, low-density lipoprotein receptor-related protein 6 (LRP6 – pS1490), GSK3 beta, Dishelleved (Dvl) 2/3, lymphoid enhancer-binding factor 1 (LEF1), transcription factor (TCF) 1/7), Hippo signaling (large tumor suppressor kinase 1 (LATS1), Mob1, macrophage stimulating protein 1 (MST1), TEA domain transcription factor 1 (TEAD), kidney and brain expressed protein (KIBRA)) and Hedgehog signaling (glioma-associated oncogene (GLI)2/3, suppressor of fused protein (SUFU)). Notably, proteins involved in cell cycle regulation (cyclin-dependent kinase (CDK) 1/2/4/6, Cyclin D1/2, p21, Rb) were also strongly elevated in NPC (Fig. 2B). For select proteins we again validated the DigiWest results with ICC. Expression levels of beta-catenin and LEF1, both critical for Wnt signaling, and of the cell cycle modulator p21 were significantly elevated in NPC compared to iPSC (Fig. 2C + D).

When investigating the differentiation process from iPSC to NPC comparing patient and healthy control-derived cells, we mainly observed strong upregulations (CTR: 89.4%, SCZ: 92.4%) and only a handful of downregulations (CTR: 10.6%, SCZ: 7.6%) for both (Additional file 5 – Figure S5). As expected, most changes were shared between CTR and SCZ, given that we already observed highly consistent expression across most samples (see Fig. 2A). Regardless, some select analytes behaved differently in SCZ vs CTR-derived cells during development, as calculated by two-factor-ANOVA (interaction effect *p* < 0.05). These (e.g. LEF1, Smurf1, p21 or ERK1/2 – pT202/Y204) are highlighted in Fig. 2E/F and are shown in more detail in Additional file 5 – Figure S6/ Additional file 1 – Table S3. Overall, these data show that the DigiWest can clearly identify cell-type specific proteomic signatures in differentiated cells thus allowing extensive analysis of cellular signaling during the differentiation process.

### SCZ iPSC show only few disease-associated alterations at the protein level

Next, we aimed to investigate disease-specific proteomic signatures at the iPSC and NPC stage, respectively. In iPSC, we found 11 (8%) analytes to be differentially expressed between CTR (*n* = 9) and SCZ (*n* = 12), with 5 becoming up- and 6 downregulated in SCZ (Fig. 3A + B). By far, the strongest effects in magnitude were observed for p53 phosphorylated at Ser15 (~ tenfold upregulation in SCZ), along with total levels of p53 (~ twofold upregulation in SCZ) (Fig. 3C). We confirmed upregulation of p53 in SCZ iPSC by ICC (Fig. 3D-E). Other de-regulated proteins showed significant, but less pronounced differences (Additional file 1 – Table S4). These data suggest that, except altered p53 expression and phosphorylation, there are relatively few prominent changes between CTR and SCZ on the iPSC level.

### Reduced differentiation capacity of SCZ iPSC into neural progenitor cells

At the NPC stage, we again compared all SCZ (*n* = 9) and control (*n* = 12) samples and identified 30 analytes (23%) significantly different between the two groups (Fig. 4A + B), three times as many as in iPSC. Of these 30, 23 proteins were upregulated, and 7 were downregulated in SCZ. Crucially, several of the eight cell type-specific markers discussed previously (see Fig. 1) were among the differentially expressed proteins. On average, SCZ NPCs displayed higher levels of Oct4 and decreased levels of MAP2, NCAM and Sox1 compared to CTR NPC, while the other markers were not affected (Fig. 4C, Additional file 5 – Figure S7). Overall, this indicates decreased differentiation efficiency of SCZ NPC. Of note, considerably inter-donor variability was observed within both CTR as well as SCZ groups regarding the expression of these proteins with clones thus contributing to the observed SCZ phenotypes to varying extent (Additional file 5 – Figure S8A-B). To evaluate if decreased differentiation efficiency of SCZ iPSC impacts early neurodevelopmental processes, we tracked neurite outgrowth of NPC for 40 h. Starting 24 h after induction of neuronal differentiation, SCZ NPC showed a reduction of mean neurite length of increasing magnitude over time compared to CTR cultures (Additional file 5 –Figure S9).

### Aberrant protein expression and dysregulated signaling pathway activity in SCZ NPC

**Fig. 5:**
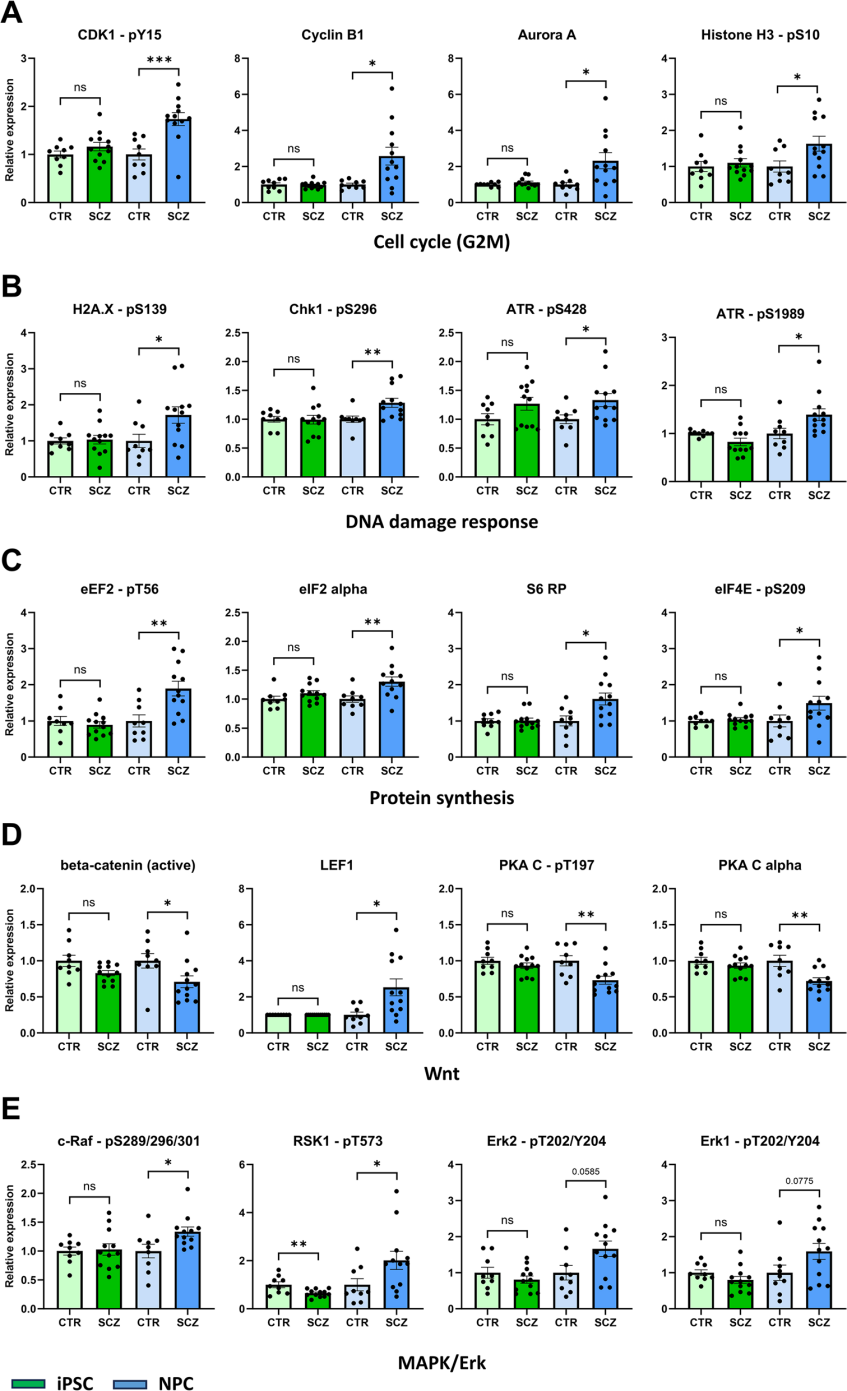
Pathway allocations of SCZ-specific proteins. **A-E**: DigiWest data (relative to CTR mean) of select proteins differentially expressed between CTR (*n* = 9) and SCZ (*n* = 12) in NPC only (see Fig. 4D), shown in direct comparison with iPSC (CTR *n* = 9, SCZ *n* = 12). Analytes are grouped based on pathway/cellular function. **A**: Cell cycle regulation, specifically G2/M phase transition, **B**: DNA damage response, **C**: Protein synthesis/translation, **D**: Wnt signaling, **E**: MAPK/Erk signaling. A complete list of all differentially expressed SCZ-specific analytes can be found in Additional file 1—Table S5. Either the Mann–Whitney test or unpaired t-test was used depending on data distribution. *p < 0.05, **p < 0.01, ***p < 0.001 or as indicated. Error bars: S.E.M

Of all de-regulated analytes, most analytes (26/30) with differential regulation were exclusive to the NPC stage (Fig. 4D) and will be discussed in detail in Fig. 5, whereas only 4 SCZ-specific effects were conserved from the iPSC stage. Besides minor changes in CDK4 and ataxia-telangiectasia mutated kinase (ATM) expression (Additional file 5 – Figure S10A), these included highly increased levels of phosphorylated p53 (Ser15) and total p53 (Fig. 4E). Noteworthy, the average magnitude of change for these two analytes was twice as high in NPC (20-fold/sixfold) as in iPSC (tenfold/threefold). Similar results were obtained with traditional Western Blotting (Fig. 4F-G). We again confirmed p53 upregulation in NPC via ICC (F ig. 4H-I), also to a greater extent than in iPSC (threefold/1.8-fold). Overall, SCZ-specific alterations of protein expression mostly appear exclusively at the NPC stage, and in the case of p53 dysregulation are more pronounced compared to iPSC.

Furthermore, several of the dysregulated analytes exclusive to NPC were phospho-variants, suggesting deregulated cellular signaling. Accordingly, most of them could be attributed to distinct signaling pathways while others serve as regulators of cellular processes (Fig. 5A-E, Additional file 1 – Table S5). Among them were cell cycle-regulating proteins such as CDK1 – pY15, Cyclin B1 and Aurora Kinase A, as well as Histone H3 – pS10 (a marker for cells undergoing mitosis) all roughly showing a twofold increase in SCZ (Fig. 5A). Total CDK1, Aurora Kinase B and Cyclin E1 (Additional file 5 – Figure S10B) were also upregulated. Notably, they all play a critical role in regulating transition into G2/M phase. Interestingly, the relative inter-donor expression levels of Cyclin B1 and Aurora A, as well as p53 and p53 – pS15 (Additional file 5 – Figure S8C), showed strikingly similar patterns to the relative expression levels of the pluripotency marker Oct4.

Additionally, we observed several alterations in the DNA-Damage-response (DDR) pathway (Fig. 5B). Histone H2A.X (S139), a primary indicator for DNA damage, as well as phosphorylation of crucial DDR proteins Chk1 (S296), and ataxia telangiectasia and Rad3-related kinase (ATR—S428 and ATR – S1989) were elevated in SCZ cells. On the other hand, phospho-ATM (S1981) showed an opposing trend for downregulation (Additional file 5 – Figure S10B).

Upregulation was also observed for several proteins and phospho-variants involved in translation such as eukaryotic elongation factor 2 (eEF2 – pT56), eukaryotic Initiation Factor 2 (eIF2) alpha, the ribosomal protein S6, as well as eukaryotic translation initiation factor 4E (eIF4E) – pS209 (Fig. 5C) along with total eEF2 and eukaryotic elongation factor 2 kinase (eEF2K) – pS366 and (Additional file 5 – Figure S10B). Also, phosphorylation of eukaryotic translation initiation factor 4E-binding protein 1 (4E-BP1, T37/S46, *p* = 0.0585) and the inhibitory site of eIF2 alpha (S51, *p* = 0.0601) showed an up- and downregulatory trend, respectively.

Moreover, we found dysregulations in Wnt signaling (Fig. 5D + Additional file 5 – Figure S10B), such as decreased active beta-catenin (non-pS33/41/45), twofold elevated LEF1, reduced protein kinase A (PKA) C (T197), total PKA C and elevated Casein kinase 1 alpha.

Finally, SCZ NPC also displayed increased phosphorylation of key MAPK proteins (Fig. 5E), namely c-Raf (S289/296/301), Erk2 (T202/Y204), Erk1 (T202/Y204) and Ribosomal S6 kinase (RSK) 1 (T573). Notably, the change of Rsk1 phosphorylation was opposite to iPSC, in which a significant reduction was observed.

Overall, protein expression patterns in NPC derived from SCZ patients suggest cell-type specific dysregulations in translation and protein synthesis, increased DNA Damage and DDR, elevated MAPK/Erk signaling, altered Wnt signaling, and higher expression of proteins governing cell cycle control, specifically those involved in regulating G2/M transition.

### SCZ NPC accumulate in G2/M phase of the cell cycle

**Fig. 6:**
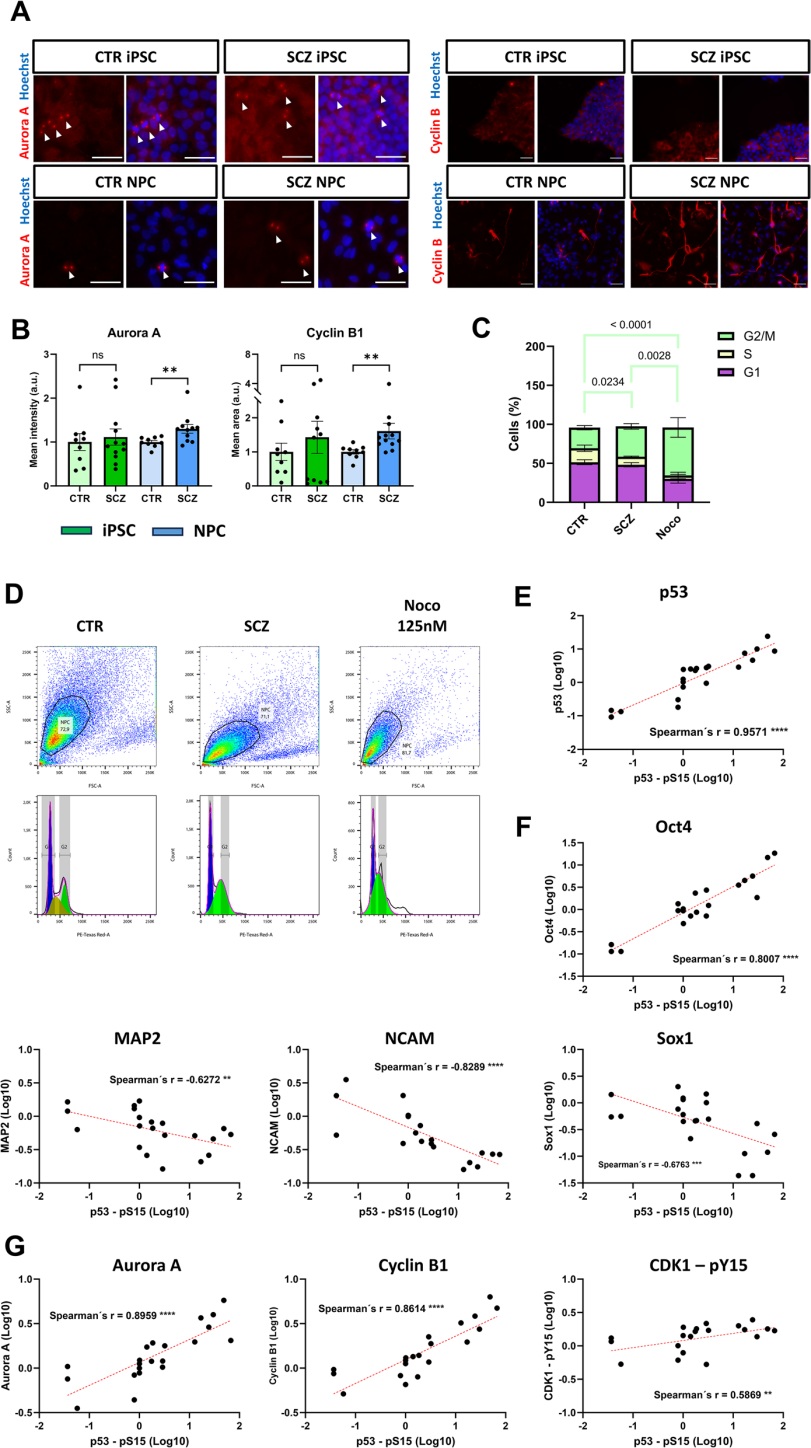
Phenotypic cell cycle alterations in SCZ NPC correlate with p53 levels. **A**: Example images of G2/M regulatory proteins Aurora A and Cyclin B1 ICC staining in CTR and SCZ iPSC and NPC obtained by high-content microscopy. Scale bars: 50 µm. **B**: Quantified ICC signal of Aurora A and Cyclin B1 expression (relative to CTR mean) in CTR (*n* = 9) and SCZ (*n* = 12) in iPSC and NPC, respectively (Mann–Whitney test). **C**: Cell phase distribution of CTR and SCZ NPC (*n* = 5–8) in percent; 2-Way ANOVA with Tukey’s multiple comparisons test. **D**: Flow-cytometry cell cycle analysis of CTR and SCZ NPC. Nocodazole was used as a positive control. **p < 0.01 or as indicated. Error bars: S.E.M. **E–G**: Correlations between p53 – pS15 and **E**: p53 (total), **F**: differentiation markers and **G**: G2/M cell phase regulators. Spearman´s r; **p < 0.01, ***p < 0.001, ****p < 0.0001. The dashed red line indicates a simple linear regression applied to each XY correlation

As upregulation of cell cycle stage-specific mediators have so far not been phenotypically linked with SCZ using iPSC-derived cell types, we aimed to investigate this aspect in more detail. ICC staining for G2/M-associated proteins Aurora A and Cyclin B1 confirmed DigiWest data and revealed an increased expression in SCZ NPC, but not iPSC (Fig. 6A + B). Furthermore, we conducted FACS analysis of propidium iodide staining in NPC to evaluate cell cycle phase distribution. Here, we observed a significantly increased percentage of cells in the G2/M phase for SCZ NPC compared to controls, while S-phase and G0/G1 phase were unaltered (Fig. 6C-D, Additional file 5 – Figure S11B). In addition, CTR and SCZ NPC showed no difference in proliferation rates, thus excluding the possibility that the rise in expression is simply due generally increased cell cycle activity of SCZ NPC (Additional file 5 – Figure S12). We also confirmed elevated Cyclin B1 expression via Western Blotting, with SCZ NPC showing an up-regulatory trend (Additional file 5 – Figure S13).

### Expression of differentiation markers and G2/M-phase proteins correlate with p53 levels

To investigate a potential link between p53 expression/phosphorylation and the observed cellular phenotypes, we performed correlation analyses in NPC across all donors among p53 – pS15, p53, differentiation markers as well as G2/M-specific cell cycle regulators (Additional file 5 – Figure S14). As p53 phosphorylation showed the most striking alteration in SCZ NPCs, its correlation analyses are shown in detail. We found a strongly significant positive correlation (Spearman´s *r* = 0.9671) with total p53 levels (Fig. 6E), indicating that p53 phosphorylation is dependent on p53 abundance. Furthermore, we observed a positive correlation with Oct4 (*r* = 0.8007) and negative correlations with MAP2 (*r* = -0.6272) and NCAM (*r* = -0.8289) and Sox1 (*r* = -0.6763), respectively (Fig. 6F), thus establishing a link between p53 and the observed reduced differentiation efficiency. p53 phosphorylation also strongly correlated with G2/M cell cycle proteins Aurora A (*r* = 0.8959) and Cyclin B1 (*r* = 0.8614), as well as moderately with CDK1 – pY15 (*r* = 0.5869, Fig. 6G).

## Discussion

Here, we report the first protein profiling analysis focusing on cellular signal transduction to study early neurodevelopmental aberrations in SCZ using iPSC-derived cell types. In a targeted proteomics approach based on the DigiWest technology, we found deregulation of protein expression mainly in NPC derived from patients with SCZ. We demonstrate regulatory alterations in cell cycle control and DNA-damage response along with impaired differentiation capacity. Additionally, we correlatively linked these aberrations to p53 expression and phosphorylation levels in diseased cells. Furthermore, we confirmed previous observations of altered protein synthesis, WNT signaling and MAPK/Erk signaling in SCZ.

The efficient differentiation of iPSC into neuronal cell types is commonly assessed via immunostaining of few selected cell type-specific markers [32]. We demonstrate that DigiWest can recapitulate characteristic pluripotency and neural progenitor marker expression equally well when compared to ICC. We show activation of several key developmental pathways such as Wnt [33], Hedgehog [34], and Hippo [25] signaling, along with changes in cell cycle regulators [35, 36]. Moreover, cells derived from SCZ patients do differ in the expression of select Wnt, MAPK and cell cycle proteins during differentiation. While other studies have also reported decreased neuronal differentiation capacity of SCZ patient-derived iPSC [37, 38], a link to changes in developmental pathway activity underlying these phenotypes has not yet been proposed. Thus overall, DigiWest is a powerful, novel tool for iPSC-based studies as it can highlight disease-associated differences which are not necessarily visible by analysis of differentiation markers alone.

NPCs have frequently been used to recapitulate SCZ pathology in vitro at early neurodevelopmental stages, as it was previously demonstrated that gene expression signatures of iPSC-derived neurons are largely conserved in NPC [9] and that protein signatures of NPC show similarities to patient postmortem brains [11]. In line with this, we mostly observed prevalent changes exclusively in NPC, indicating that most alterations and dysfunctions in pathway regulation occur in differentiated cells, but are not yet present in iPSC. For instance, we provide evidence for regulatory changes of protein synthesis, MAPK signaling and Wnt signaling in SCZ NPC. In support of our findings, several reports using iPSC models of SCZ indicate changes in translation and protein synthesis [6, 8, 11] as well as altered WNT signaling in SCZ NPC [39], neurons [16] and patients [14].

Notably, we did find highly elevated expression levels of p53 and its phosphorylated variant p53-pS15 in SCZ already at the iPSC stage. p53 is a tumor suppressor protein with central roles in regulating cellular responses to genomic alterations and DNA damage along with controlling of the cell cycle and apoptosis. Importantly, p53 is only phosphorylated at Ser15 in response to DNA damage [37, 38]. Furthermore, this phosphorylation event stabilizes p53 levels by reducing interaction of p53 with its negative regulator mouse double minute 2 homolog (MDM2) [40]. p53 is mainly associated with tumorigenesis but also plays a role in brain development, neural stem cell regulation [41], and in several nervous system diseases including SCZ, autism, Parkinson´s disease, Alzheimer´s disease or epilepsy [42]. *TP53* has been identified as a candidate risk gene in SCZ in gene association [43] as well as case–control/family studies [44] have specifically associated genetic variation in *TP53* with SCZ. Further support for an involvement of p53 is given by extensive evidence showing a negative correlation between SCZ and cancer incidence [22, 45, 46].

It has been hypothesized that p53 upregulation in stem cells due to genotoxic stress impairs pluripotency and might lead to unwanted, unspecific differentiation [39]. This aspect could at least in part explain our finding of reduced differentiation efficiency of SCZ iPSC upon neural induction and the resulting lower state of maturity of SCZ NPC, as indicated by higher expression of OCT4 and lower expression of MAP2, NCAM and SOX1. Crucially, for MAP2 and NCAM, transcriptomic profiling of a different set of SCZ patient-derived NPC revealed similar effect [9]. The lower maturation state of SCZ NPC might impact the efficiency of early neurodevelopmental processes, as reflected in a reduction of neurite outgrowth which we and others [9, 47] have observed in SCZ. In accordance with our results, decreased MAP2 expression along with alterations in pathways governing neuronal differentiation have also been reported in SCZ iPSC-derived organoids [23, 48]. Moreover, impaired neuronal differentiation efficiency has been described for 22q11.2 deletion iPSC [38], a CNV associated with SCZ, and patient-derived glutamatergic neurons [37]. It can also be hypothesized that impaired neuronal maturation could influence other key neurodevelopmental processes such as neuronal synapse formation, which is impaired in SCZ [21]. This however needs further investigation. Importantly, the strong up-regulatory trend of p53 was the only notable SCZ-specific alteration present in both iPSC and NPC, highlighting its importance in the context of differentiation.

In addition, our results indicate that SCZ NPC show increased levels of damaged DNA and associated DNA-damage response. While the overarching picture of how DNA damage is implicated in neurogenesis and SCZ is still incomplete, it has been associated with the disease [49, 50]. For instance, genetic alterations in DNA repair enzymes were linked to SCZ [51] or can induce behavioral changes in mice [52]. A recent transcriptomic study of postmortem SCZ brain tissue also uncovered increased DNA damage repair [53]. Moreover, mass spectrometry analysis of SCZ-derived neural stem cells found alterations in pathways related to mitochondrial function, metabolic activity and DNA repair [54]. Lastly, reactive oxygen species and oxidative stress were shown to be elevated in SCZ NPC [9, 55] and animal models [56]. Thus, our data provide further evidence for a significant implication of DNA damage and repair mechanisms in SCZ pathology. Interestingly, we demonstrate that SCZ NPC display higher levels of G2/M-associated proteins, where cells are checked for DNA damage before cell cycle progression [57]. While we did not observe changes in basal proliferation rate of NPC, our FACS analysis showed a greater proportion of SCZ cells in G2/M phase—it is tempting to speculate that SCZ NPC become stuck in the G2/M phase, potentially leading to delayed cell cycle exit/progression during differentiation. Crucially, p53 can cause G2/M arrest in response to DNA damage [58]. Based on our data, we cannot infer whether cells arrest (and later attempt to recover) or exit the cell cycle temporarily; accordingly, cell cycle arrest has been described as a highly dynamic molecular state [59]. An association of DNA damage and G2/M phase in SCZ has thus far not been reported, although studies have shown alterations in cell cycle control in SCZ models [54, 60, 61] and the relevance of cell phase regulation and progression in neurodevelopment [62]. Most prior studies on SCZ proteomics/transcriptomics/genomics use shotgun approaches, merely reporting SCZ-specific changes on pathway-association level. With DigiWest being a targeted approach, we were able to focus on more detailed mechanisms of pathway activation – also by additionally measuring phosphorylation.

We identified such coherent proteomic changes while confirming previous findings from other omics approaches despite a small cohort size and notable inter-patient variability. Moreover, the four patients had diverse genetic backgrounds and disease manifestations, thus covering various clinical phenotypes, which is helpful in studying multifaceted diseases such as SCZ. Notably, for some of the measured analytes, we detected strong inter-donor variability, likely due to differences in the genetic background of each individual, which was previously shown to be the largest source of variability in a proteomic study on iPSC-derived astrocytes [63]. In addition, there are several other potential sources of inter-donor variability, including donor sex [64] and age, as exonic mutations can accumulate over time [65]. In our dataset, patient line SCZ1 displayed the strongest phenotype for key observations (highest p53 expression, lowest differentiation capacity, highest G2/M proportion), while the SCZ2 patient line showed a more CTR-like phenotype in certain aspects (also see Additional file 5 – Figure S8A). This needs to be kept in mind when interpreting the data as it might reflect the phenotype of certain subgroups of patients only. These limitations could be accounted for in future studies employing a larger number of patients to disentangle the relationship between individual sources of variation and SCZ phenotypes.

Although the aspects discussed above (p53, cell cycle, differentiation efficiency) all have individually been shown to be implicated in SCZ, our correlation data for the first time provide a possible connection between them. It can be speculated that patient-derived progenitor cells acquire DNA damage during early development and deal with it inadequately, causing cell cycle arrest or delay. This could hamper differentiation efficiency, resulting in less mature NPC and eventually neurons, which might contribute to the developmental defects associated with SCZ. In future studies it would be of interest to investigate whether our observed proteomic alterations and association with p53 will be confirmed in larger cohorts to further deepen the understanding of neurodevelopmental failure in SCZ from a mechanistic standpoint.

## Conclusion

Using a high-throughput targeted proteomics approach, we uncover aberrant protein expression signatures in SCZ patient-derived NPC. Moreover, diseased cells displayed regulatory changes in cell cycle control along with impaired differentiation capacity. Using our proteomic data, we demonstrate a potential link of these phenotypes to aberrant p53 expression and phosphorylation. We ultimately hypothesize a potential interplay of these disease-specific alterations affecting the differentiation process during early neurodevelopment, which could be mechanistically implicated in the manifestation of developmental alterations occurring in SCZ. Further studies could explore the significance of our findings in larger cohorts using patient-derived neuronal cell types in iPSC-based model systems. Our study not only demonstrates the importance and significance of stem cell-based model systems and protein-based analytics in psychiatric disorders such as SCZ but also highlights our patho-mechanistic findings as accelerators in the search for potential drug targets.

## Supplementary Information

Additional file 1: Supplementary Tables

Additional file 2: DigiWest Antibodies

Additional file 3: DigiWest Raw Data

Additional file 4: DigiWest Normalized Data

Additional file 5: Supplementary Figures

## Data Availability

All DigiWest-related raw and normalized data generated or analyzed during this study are included within the article (and its supplementary information files). All other datasets (Western Blot, ICC and FACS) used and/or analyzed during the study are available from the corresponding author on reasonable request. All other datasets (Western Blot, ICC and FACS) used and/or analyzed during the study are available from the corresponding author on reasonable request.

## Abbreviations

4E-BP1: Eukaryotic translation initiation factor 4E-binding protein 1
ATM: Ataxia-telangiectasia mutated kinase
ATR: Ataxia telangiectasia and Rad3-related kinase
CDK: Cyclin-dependent kinase
CTR: Control
DDR: DNA damage response
eEF2: Eukaryotic elongation factor 2
eIF2: Eukaryotic initiation factor 2
eIF4E: Eukaryotic translation initiation factor 4E
ERK: Extracellular signal-regulated kinases
FACS: Fluorescence-activated cell sorting
GLI: Glioma-associated oncogene
GSK3 beta: Glycogen synthase kinase-3 beta
ICC: Immunocytochemical staining
iPSC: Induced pluripotent stem cells
KIBRA: Kidney and brain expressed protein
LATS1: Large tumor suppressor kinase 1
LEF1: Lymphoid enhancer-binding factor 1
LRP6: Low-density lipoprotein receptor-related protein 6
MAP2: Microtubule-associated protein 2
MAPK: Mitogen-activated protein kinase
MDM2: Mouse double minute 2 homolog
MST1: Macrophage stimulating protein 1
NCAM: Neural cell adhesion molecule
NPC: Neural progenitor cells
PAX6: Paired-box protein Pax-6
PKA: Protein kinase A
RSK1: Ribosomal S6 kinase 1
RT: Room temperature
SCZ: Schizophrenia
SOX1: SRY-Box Transcription Factor 1
SOX2: SRY-Box Transcription Factor 2
SUFU: Suppressor of fused protein
TCF1/7: Transcription factor 1/7
TEAD1: TEA domain transcription factor 1
