# Supplementary material for: Patient iPSC-derived neural progenitor cells display aberrant cell cycle control, p53, and DNA damage response protein expression in schizophrenia
Source: BMC Psychiatry. 2024 Oct 31;24:757. doi: 10.1186/s12888-024-06127-x (PMC11526604; doi:10.1186/s12888-024-06127-x)
Supplement: Supplementary file 1 — Additional file 1: Supplementary Tables [file 12888_2024_6127_MOESM1_ESM.docx]

# Additional file 1: Supplementary tables

## Table S1: iPSC line information

## Table S2: Antibody list for immunocytochemistry

## Table S3: Interaction effect – cell type and disease state

## Table S4: CTR versus SCZ in iPSC

## Table S5: CTR versus SCZ in NPC
