# Supplementary material for: Patient iPSC-derived neural progenitor cells display aberrant cell cycle control, p53, and DNA damage response protein expression in schizophrenia
Source: BMC Psychiatry. 2024 Oct 31;24:757. doi: 10.1186/s12888-024-06127-x (PMC11526604; doi:10.1186/s12888-024-06127-x)
Supplement: Supplementary file 5 — Additional file 5: Supplementary Figures [file 12888_2024_6127_MOESM5_ESM.docx]

# Additional file 5: Supplementary figures

## Figure S1: Entire DigiWest data set

**Figure S1:** Heatmap and Hierarchical Cluster analysis of entire DigiWest data set including all samples (n = 42). Clustering was performed using Euclidian Distance and complete linkage.

## Figure S2: iPSC versus NPC comparison

**Figure S2:** Volcano plot of DigiWest iPSC (n = 21) and NPC (n = 21) comparison. Significantly upregulated proteins are shown in red, downregulated proteins in blue with marker proteins being annotated; Wilcoxon Test, p<0.001. Significantly different analytes with FC < I1I were excluded.

## Figure S3: Uncropped Western Blot mimics of differentiation markers

**Figure S3:** Uncropped Western Blot mimic images (grayscale) of marker proteins and beta-Actin corresponding to **Figure 1B** (exemplarily shown for one differentiation only).

## Figure S4: Quantification of ICC markers

**Figure S4**: Exemplary quantification of Oct4, MAP2, Vimentin and NCAM ICC signals. Corresponding representative example images are shown in Figure 1C (iPSC n = 21, NPC n = 21). Data are shown relative to mean iPSC signal; Mann-Whitney test or unpaired t-test depending on data distribution. *p < 0.05, ****p < 0.0001. Error bars: S.E.M.

## Figure S5: iPSC versus NPC – disease allocation

**Figure S5:** Heatmaps and Hierarchical Cluster (HCL) analyses corresponding to Figure 2E/F, respectively showing analytes significantly different between **A:** CTR iPSC (n = 9) and NPC (n = 9) and **B:** SCZ iPSC (n = 12) and NPC (n = 12); Wilcoxon Test, p<0.01. The respective percentage of differential expression is also indicated along with number of analytes significantly different across the respective groups.

## Figure S6: Interaction effect of cell type and disease state

**Figure S6:** DigiWest data (AFI) of analytes with significant interaction effect (p<0.05) or trend (p > 0.05 < 0.08) between cell type and disease allocation (see SupplementaryTable2); 2-way-ANOVA with Tukey´s multiple comparisons test. CTR iPSC (n = 9), SCZ iPSC (n = 12), CTR NPC (n = 9), SCZ NPC (n = 12). *p < 0.05, **p < 0.01, ***p < 0.001, ****p < 0.0001. Error bars: S.E.M.

## Figure S7: Differential expression of marker proteins in CTR and SCZ

**Figure S7:** DigiWest data (relative to CTR mean) of Vimentin, Sox2, Pax6 and Nestin expression in CTR (n = 9) and SCZ (n = 12) iPSC and NPC, respectively; Mann-Whitney test.

## Figure S8: Phenotypes of individual clones and corresponding DigiWest data

**Figure S8:** **A:** Principal component analysis (PCA) of DigiWest dataset including all individual clones. PC = principal component; proportion of variance as indicated. **B-C:** DigiWest data (relative to CTR mean) of all individual CTR and SCZ clones for **B:** differentiation/neuronal markers Oct4, MAP2, NCAM and Sox1 as well as **B:** p53 – pS15, p53 (total), Cyclin B1 and Aurora A. Indicated significance is taken from statistics performed in **Figure 4C, Figure 4E and Figure 5A**. Each bar represents n = 3 per clone. *p < 0.05, **p < 0.01 or as indicated. Error bars: S.E.M.

## Figure S9: Neurite outgrowth assay (NPC)

**Figure S9:** **A:** Brightfield images of CTR and SCZ NPC with outgrowing neurites (purple) at 0h, 16h and 32h after neural induction. **B:** Neurite outgrowth assay of CTR and SCZ NPC. Analysis of neurite length relative to number of cell body clusters over a time period of 40 h (CTR: n = 24, SCZ: n = 32 per time point). 2-way-ANOVA with Tukey´s multiple comparisons test. *p < 0.05, **p < 0.01, ***p < 0.001, ****p < 0.0001. Error bars: S.E.M.

## Figure S10: Stage-conserved and additional NPC-specific disease effects

**Figure S10: A:** DigiWest data (relative to CTR mean) of CDK4 and ATM for SCZ-specific effects consistent at the iPSC and NPC stage and of **B:** further relevant analytes with respective pathway allocation not shown in Figure 5. CDK1 - total, Aurora B, Cyclin E1 (G2/M/cell cycle), ATM – pS1981 (DNA damage response), eEF2, eEF2k – pS366, 4E-BP1 – pT37/41, eIF2 – pS51 (protein synthesis), CK1 (Wnt signaling); CTR iPSC (n=9), SCZ iPSC (n=12), CTR NPC (n=9), SCZ NPC (n=12). Either the Mann-Whitney test or unpaired t-test was used depending on data distribution. *p < 0.05, **p < 0.01, ***p < 0.001 or as indicated. Error bars: S.E.M.

## Figure S11: FACS analysis (NPC)

**Figure S11:** **A:** Gating strategy for FACS cell cycle assay. **B:** Cell phase distribution of individual CTR and SCZ clones. Indicated significance is taken from statistics performed in **Figure 6C**. Each bar represents > 15,000 events recorded from 1-2 wells per line. p-value as indicated. Error bars: S.E.M.

## Figure S12: Proliferation assay (NPC)

**Figure S12:** Proliferation assay showing no difference in growth rate between CTR and SCZ NPC over a time period of 72 h; Mixed-effects model with Šídák's multiple comparisons test, p = 0.1297. CTR: n=9 per time point, SCZ: n=12 per time point. Data was obtained from three independent experiments per line. Error bars: S.E.M.

## Figure S13: Cyclin B1 Western Blot

**Figure S13: A:** Western Blot of Cyclin B1 expression in NPC. **B:** Quantified Western Blot signals from A relative to CTR mean. Intensities were normalized to beta-Actin signal; CTR n = 6, SCZ n = 8, Mann-Whitney test. p-value as indicated. Error bars: S.E.M.

## Figure S14: Correlation matrix

**Figure S14:** Correlation matrix (Spearman´s r) of p53 – pS15, p53, Oct4, NCAM, MAP2, Sox1, Cyclin B1, AURKA and CDK1 – pY15 expression levels in NPC (n = 21). DigiWest signals are shown as Log10 of CTR mean-centered AFI. Positive correlations are indicated in blue, negative correlations in red.

## Figure S15: Uncropped Western Blot images

**Figure S15:** Uncropped Western Blot images of NPC for p53 – pS15, total p53, Cyclin B1 and beta-Actin corresponding to **Figure 4G** and **Additional file 5 – Figure S13A**. For each clone, NPC were obtained from two separate differentiations. Beta-Actin is shown as reference.
